# Squamous cell carcinoma of the paranasal sinuses: cutaneous metastases with bone involvement

**DOI:** 10.1590/0100-3984.2016.0113

**Published:** 2018

**Authors:** Bruno Niemeyer de Freitas Ribeiro, Bernardo Carvalho Muniz, Tiago Medina Salata, Diogo Goulart Corrêa, Edson Marchiori

**Affiliations:** 1 Instituto Estadual do Cérebro Paulo Niemeyer, Rio de Janeiro, RJ, Brazil.; 2 Hospital Casa de Portugal - 3D Diagnose, Rio de Janeiro, RJ, Brazil.; 3 Universidade Federal do Rio de Janeiro (UFRJ), Rio de Janeiro, RJ, Brazil.

Dear Editor,

In 2014, a 29-year-old female, diagnosed with squamous cell carcinoma of the floor of the frontal sinus, was submitted to surgical excision of the lesion and to radiotherapy. The following year, there was recurrence of the lesion, after which complete remission was not achieved. In 2016, she developed multiple vegetative, ulcerated lesions affecting the scalp, some provoking discrete bone involvement (Figures 1A and 1C). Magnetic resonance imaging (MRI) revealed expansile, heterogeneous lesions that showed predominantly hypointense signals in T1-weighted sequences and isointense or hypointense signals in T2-weighted sequences, with heterogeneous gadolinium enhancement and restricted diffusion (Figures 1B and 1D), aspects similar to those of the primary tumor. These findings, taken together with the clinical history, were suggestive of secondary neoplastic involvement of the skin, which was confirmed by the histopathological study.

**Figure 1:**
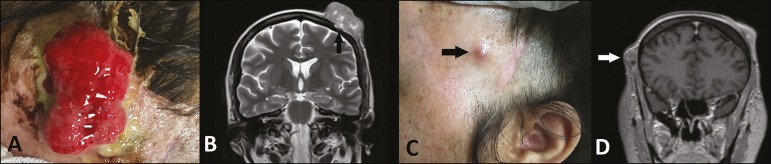
**A**: Vegetative, ulcerated lesion affecting the scalp. **B**: Coronal T2-weighted MRI sequence showing an expansile lesion affecting the parietal region, with a predominantly isointense or hypointense signal, provoking discrete bone involvement (arrow). **C**: Synchronous vegetative lesion affecting the temporal region (arrow). **D**: Coronal T1-weighted MRI sequence showing a synchronous lesion, with heterogeneous enhancement, in the right temporal region (arrow).

Recent studies in the radiology literature have emphasized the importance of MRI in improving the diagnosis of lesions of the head and neck^1-4^. Squamous cell carcinoma is derived from suprabasal keratinocytes. The incidence of the disease is highest in individuals between 50 and 70 years of age, and it affects men more often than women. Risk factors depend on the site, cigarette smoking and alcoholism being the main risk factors in cases of mucosal lesions, whereas the main risk factors in cases of cutaneous lesions are ultraviolet radiation, chronic ulcers, and fistulas. Among malignant neoplasms of the head and neck, squamous cell carcinoma is the most common, accounting for 5% of all cases of cancer^3^. The metastatic dissemination of such carcinomas is typically to the lymph nodes, although the lungs, bones, and liver can also be affected^5^.

The frequency of metastases to the skin is rare, ranging from 0.7% to 9%^6^, such metastases occurring mainly in advanced-stage lung and breast cancers, predominantly affecting the scalp, neck, forearm, thigh, or penis^6,7^.

To our knowledge, there have been no studies discussing the imaging characteristics of squamous cell carcinoma metastases to the skin. In the case presented here, the lesions were similar to the primary tumor, with hypointense signals in T1-weighted sequences and isointense or hypointense signals in T2-weighted sequences, as well as heterogeneous gadolinium enhancement and restricted diffusion. Recent studies highlight the use of diffusion-weighted sequences in the evaluation of head and neck lesions, showing that apparent diffusion coefficient values below 1.22 × 10^−3^ mm^2^/s are suggestive of malignancy^3,4,8^. In our case, the apparent diffusion coefficient value was 0.78 × 10^−3^ mm^2^/s, thus corroborating those previous findings.

The differential diagnosis of cutaneous lesions is extensive, including hemangiomas, pilomatrixomas, tuberculosis, leishmaniasis, lymphomas, and sarcomas. Although imaging methods can help in distinguishing among the causes, the diagnosis in typically made through histopathological analysis.

Cutaneous metastases are uncommon and do not present specific imaging characteristics. They should nevertheless be considered among the diagnostic possibilities in cases of cutaneous lesions, particularly when there is a known history of neoplasia.

## References

[1] Niemeyer B, Marchiori E (2015). Giant pilomatrixoma: conventional and diffusion-weighted magnetic
resonance imaging findings. Radiol Bras.

[2] Niemeyer B, Salata TM, Antunes LO (2015). Desmoplastic fibroma with perineural spread: conventional and
diffusion-weighted magnetic resonance imaging findings. Radiol Bras.

[3] Niemeyer B, Bahia PRV, Oliveira ALVSM (2012). Lethal midline granuloma syndrome: a diagnostic
dilemma. Radiol Bras.

[4] Gonçalves FG, Ovalle JP, Grieb DFJ (2011). Diffusion in the head and neck: an assessment beyond the
anatomy. Radiol Bras.

[5] Calhoun KH, Fulmer P, Weiss R (1994). Distant metastases from head and neck squamous cell
carcinomas. Laryngoscope.

[6] Sawali H, Yunus MRM, Ai OC (2010). Cutaneous metastases from nasopharyngeal carcinoma: a rare
manifestation. PJOHNS.

[7] Luk NM, Yu KH, Choi CL (2004). Skin metastasis from nasopharyngeal carcinoma in four Chinese
patients. Clin Exp Dermatol.

[8] Wang J, Takashima S, Takayama F (2001). Head and neck lesions: characterization with diffusion-weighted
echo-planar MR imaging. Radiology.

